# Editorial Comment: Anxiety and depression associated with a positive prostate biopsy result: A comparative, prospective cohort study

**DOI:** 10.1590/S1677-5538.IBJU.2019.0719.1

**Published:** 2020-09-02

**Authors:** André Luiz Lima Diniz

**Affiliations:** 1 Departamento de Urologia Instituto Nacional do Câncer Rio de JaneiroRJ Brasil Departamento de Urologia, Instituto Nacional do Câncer – INCA, Rio de Janeiro, RJ, Brasil

## COMMENT

Developed in the 1970s, transrectal ultrasound of the prostate applied to biopsy of that organ started to be used more widely in the 1980s ( 1 , 2 ). In that decade, the use of this technology among renowned teams allowed great knowledge to be acquired about prostate anatomy, the procedure and complications ( 3 , 4 ).

Much has been changed since then, devices have developed substantially, new imaging methods have been added, protocols and guidelines have been outlined for the proper use of this diagnostic tool. So, after more than 30 years of publications, the most skeptical of urologists would say that this science is exhausted, and no other paper could emerge on this topic.

With great satisfaction, the International Brazilian Journal of Urology once again brings to our dear readers an assessment of prostate biopsy guided by ultrasound from a different perspective. In previous editions, new data about complications of the procedure ( 5 ) and evaluation of the pain pattern in different methods of sample acquisition ( 6 ) were presented. In this issue we bring you an article that discusses the mood disorders associated with biopsy and positive results.

The wide access and use of screening methods, made part of our actions somewhat mythical. The lack of real understanding about PSA and all the taboos inherent to digital rectal examination, together are enough to create a fog of doubt and fear among men in the age group for screening.

In other areas of oncology, where screening methods are possible, patients also face similar challenges before diagnosis; and once it is done, a storm of dubious thoughts sets in their minds and souls. Among women, confirmation of a breast cancer or a cervical and its treatment may impact on self-image and sexual concerns which may lead to mood disorders and worsening quality of life ( 7 - 10 ). Likewise, a positive diagnosis through colonoscopic screening for malignancies can bring countless questions to the patient who, without assertive information or even without adequate support, can develop anxiety or depression ( 11 ).

In the present study Sefik et al. demonstrated that, before biopsy, levels of anxiety and depression measured were correlated with serum levels of PSA. Demonstrating one of the negative effects that a “simple blood test” could have on individuals. And they show us more: just after biopsy, those symptoms disappeared; this is related to the good practice adopted in that department, where the patient is well oriented on the technique of the procedure just before it ( 12 ).

In Rio de Janeiro, Brazil, at the National Cancer Institute - INCa - a branch of the Urology Department, the Prostate Cancer Diagnosis Center - CDCP –welcomes referral patients from all over the state, whose biopsy was requested in primary care by a family and community physician or even by urologists serving in public settings. Understanding the burden of expectations related to that referral, our Center proposes a multi-professional approach to the patient and his family.

The first contact happens in consultation with a urologist experienced in oncology, in which the biopsy indication is re-evaluated and potential complicating factors (such as previous comorbidities, urinary infections and the use of medications) are checked. Once the intervention is safely indicated, our urologist explains to the patient the reason for the need for the procedure, possible complications and preoperative care. Moments later, a second opportunity to explain and clarify doubts occurs, but now in consultation with a nurse who answers the remaining questions and reinforces the care prior to the procedure, in addition to providing antibiotic and glycerin suppository, advising on their use.

The second multi-professional contact occurs on the day of the biopsy; the initial approach is made by the nurse who verifies that the recommendations were made properly and then is evaluated by the anesthesiologist who guides the family and the patient on the procedures to be followed. Our biopsy is performed by an interventional radiologist with the patient monitored under total venous sedation. Before being discharged, the patient and his companion are advised on post-procedure care and informed of the return date to receive the biopsy result directly from our urologists.

In this third visit to the CDCP, it is recommended that the family member be present again, in the expectation that, in the face of a positive result, the patient will have a supportive figure at that moment. The various possible approaches are explained according to the result of the examination and the risk of metastasis; the patient is referred by the state regulation system to the units where he can be treated.

All the zeal described above follows the idea that information can overcome fear. A well-oriented patient is armed to face all the challenges that will come over the months after diagnosis. Sefik and his team also believe that anxiety can be relieved with a good explanation.

Anxiety and depression are risk factors for non-adherence to treatments that will be proposed ( 13 ), but in face of so many issues to be solved, symptoms of mood disorders can slowly set in; so those diseases can be neglected and ultimately untreated. It becomes clear that diagnosing and treating anxiety and depression in patients diagnosed with prostate cancer is fundamental to improve quality of life and reduce mortality.

In a study published by Dept. of Psychiatry and Behavioral Sciences, Memorial SloanKettering Cancer Center, researchers evaluated several types of neoplasms; among patients with prostate cancer the prevalence of mixed symptoms of anxiety and depression was 10.2%. It was demonstrated that mixed anxiety/depression symptoms is associated with poorer psychosocial and treatment outcomes, worse quality of life, poorer adherence to treatment, slower recovery and greater suicide risk ( 14 ).

A meta-analysis of data from 16 prospective cohort studies was carried out by researchers from University College London, Edinburgh University and the University of Sydney. Researchers have shown that people with highest levels of psycological distress, compared to those with lowest levels of mental disturbance, were more likely die from prostate cancer (HR 2.42, 95% CI 1.29 to 4.54). Considering all the neoplasms evaluated, individuals with the highest levels of mental distress were 32% more likely to have died due to their oncological condition (HR 1.32, 95% CI 1.18 to 1.48) ( 15 ).

Sefik et al. ( 12 ) used relevant tools to assess signs of mood disorders; researchers went to great lengths to bring us data faithfully. The number of interviewed individuals impressively exceeded the calculated sample size. All this effort and diligence combined with an unique statistical refinement makes this material a delight for those who seek to combine science and daily practice.

So once we understand that a patient in route of prostate cancer diagnosis is susceptible to develop serious mood disorders which will impact future results, it should change our behavior to seek ways to help him to face this acute stressor through proven effective means. Singing sweet songs with those “Three little birds” will not make every little thing be alright. Much more must be done. It is necessary to go beyond blind positivity.

In the privacy of your office, empathy translated into good guidance seems to represent the sincerest behavior on the part of the urologist. When possible, the follow-up with human resources that could help the patient in dealing with the various discomforts to come will represent great relief and a sense of support.

Once diagnosed with cancer, people are expected to mobilize psychosocial resources to adaptively deal with the level of stress resulting from oncological disease, this strategy is called coping. The two most common forms of classification of coping are positive and negative. Positive coping focuses on approaching the problem (for example, consulting with others for advice – a pro-social coping or seeking support in faith – a religious coping). In the other direction, negative coping is marked by avoiding (for example, ignoring the problem – passive coping) or other poor adaptation efforts (for example, self-blaming or drug abuse) ( 16 , 17 ).

In a systematic review of qualitative studies, Spendelow and colleagues identified that men with prostate cancer access a wide variety of coping methods and some them may reflect the dominant masculine scripts such as ‘self-reliance’ and ‘emotional control’; approaches likely to be undesirable given the potential link between restricted emotional expression and psychological adjustment (18–20).

Roesch et al. presents in a meta-analytic review that men with prostate cancer who used positive coping methods: approach, problem-focused, and emotion-focused coping were healthier both psychologically and physically ( 19 ). Moreira-Almeida and colleagues found evidence in their well-structured meta-analysis that religious involvement is usually associated with better mental health ( 21 ). In a qualitative study, Viser et al. assessed the role of spiritual significance in dealing with cancer and noted that faith coping represents a field where care providers should be aware of the different ways in which the patient’s previous beliefs and experiences influence their current adaptation ( 22 ).

In this sense, there are those who will direct their energies towars the improvement of virtues and other will be inclined to addictions, demonstrating that leaving the patient adrift can be harmful. Professional intervention would represent good winds during storms.

The paper brought by IBJU showed us that the last point of the investigation line - suspicion / biopsy / diagnosis - is the most impactful stressor event in the process. So once prostate cancer is confirmed, regarding mood disorders, considering referral to integrated psychology and psychiatry teams will promote necessary attention in this regard. As for social welfare, especially if the financial issue afflicts the patient, social workers can guide our patients to access the rights guaranteed in the constitution of each country and region. Attending religious services, may help patients to reset sense of meaning in life.

Again, the exercise of empathy through the explanation to the patient about the phases of the procedure by the team approach (nurse, urologist, and anesthesiologist) is certainly essential for him to feel properly welcomed.
